# Febuxostat ameliorates muscle degeneration and movement disorder of the dystrophin mutant model in *Caenorhabditis elegans*

**DOI:** 10.1186/s12576-023-00888-y

**Published:** 2023-11-10

**Authors:** Sawako Yoshina, Luna Izuhara, Rei Mashima, Yuka Maejima, Naoyuki Kamatani, Shohei Mitani

**Affiliations:** 1 Department of Physiology, Tokyo Women’s Medical University School of Medicine, 8-1, Kawada-Cho, Shinjuku-Ku, Tokyo, 162-8666 Japan; 2 Tokyo Women’s Medical University School of Medicine, 8-1, Kawada-cho, Shinjuku-ku, Tokyo, Japan; 3 Stagen. Co. Ltd., 4-11-6, Kuramae, Taito-Ku, Tokyo 111-0051, Japan

**Keywords:** *Caenorhabditis elegans*, Dystrophin mutation, Febuxostat, Muscle degeneration, Movement disorder

## Abstract

Duchenne muscular dystrophy (DMD) is an inherited disorder with mutations in the dystrophin gene characterized by progressive muscle degeneration and weakness. Therapy such as administration of glucocorticoids, exon skipping of mutant genes and introduction of dystrophin mini-genes have been tried, but there is no radical therapy for DMD. In this study, we used *C. elegans* carrying mutations in the *dys-1* gene as a model of DMD to examine the effects of febuxostat (FBX). We applied FBX to *dys-1* mutant animals harboring a marker for muscle nuclei and mitochondria, and found that FBX ameliorates the muscle loss. We next used a severer model *dys-1; unc-22* double mutant and found the *dys-1* mutation causes a weakened muscle contraction. We applied FBX and other compounds to the double mutant animals and assayed the movement. We found that the administration of FBX in combination of uric acid has the best effects on the DMD model.

**Supplementary Information:**

The online version contains supplementary material available at 10.1186/s12576-023-00888-y.

## Background

Duchenne muscular dystrophy (DMD) is an X-linked muscular disease with mutations in the dystrophin gene [1]. The disease is characterized by the progressive atrophy of skeletal muscle and is often associated with dilated cardiomyopathy and autism spectrum disorder [2–5]. Skeletal muscle atrophy is caused by repetition of muscle cell damage and repair, eventual death and replenishment by satellite cells and skeletal muscle stem cells [6]. Muscle atrophy is caused by the depletion of satellite cells due to excessive loss of muscle cells [6]. The mechanisms how muscle cells are damaged involve fragile sarcolemma caused by the absence of dystrophin, which leads to muscle weakness in response to mechanical stress [7]. During muscle deterioration, muscle cells undergo mitochondrial myopathy [8].

Because the disease has been considered intractable, many researchers have endeavored to find cures. For example, glucocorticoid administration, exon skipping of the mutated gene and introduction of the dystrophin mini-gene have been used and or are being tried [5, 9], but the disease remains fatal. Although nucleotide therapy appears promising, it may not be enough considering that patients often suffer from brain and heart symptoms. Therefore, in addition to nucleotide therapy, it is desirable to develop therapies in which compounds are administered via the oral pathway.

Previously, we successfully used the anti-gout drug febuxostat (FBX) to challenge the slowdown of sarcopenia associated with aging in the nematode *C. elegans* [10]. The compound inhibits the degradation of hypoxanthine to xanthine and to uric acid [11]. The compound relieves the load to mitochondria and protects mitochondria from deterioration during aging. Because skeletal muscle cells in DMD patients may share some mechanistic characteristics with aging sarcopenia, we investigated the effects of FBX on a nematode animal model of DMD with dystrophin deletion mutations. The aim of this work was to examine whether FBX can ameliorate the phenotypes of the DMD model in the nematode *C. elegans*.

## Materials and methods

### Nematode strains

*C. elegans* strain N2 worms were used as wild-type animals. The worms were grown at 20 °C under well-fed conditions using standard methods [12]. The strain carrying *dys-1(tm4402)* was obtained from a UV/TMP-mutagenized library as described previously [13]. These were identified via PCR amplification with primers spanning the deletion region of t*m4402*, as described previously [13, 14]. The primers used for PCR genotyping were as follows: 5ʹ- CGACCAATCTTGAAGTGGCT-3ʹ and 5ʹ- GCTCTGCAAATCCCGCCACA-3ʹ (*tm4402*, 1st round); 5ʹ- GGCTCGAGCTCATGGGAAAG-3ʹ and 5ʹ- CGCCCACAGCACATCATCAG-3ʹ (*tm4402*, 2nd round). The mutant was backcrossed twice with N2 before use.

The Caenorhabditis Genetics Center provided the *unc-22(e66)* sample.

All assays were performed on kanamycin (Km)-supplemented NGM plates with UV-irradiated OP50 as food unless otherwise indicated. UV treatment of bacteria was performed as described previously [15].

### Mitochondrial imaging and nuclear imaging

*ccIs4251 (Pmyo-3::Ngfp-lacZ; Pmyo-3::Mtgfp)*, which has GFP fusion proteins localized to the body wall muscle mitochondria and nuclei, was used for this study. For synchronized worms, eggs were collected by bleaching transgenic (Tg) animals (*ccIs4251*) and reared at 20 °C on OP50 normally-seeded NGM plates (Day 0). After 36 h, the worms were transferred to NGM plates with FBX (0, 5 or 10 µg/ml) and Km. UV-irradiated OP50 was used as food. After Day 4, we replated every day until the worm stopped laying eggs. On Days 12 after bleaching, worms were anesthetized by placing M9 buffer with a drop of 50 mM sodium azide on the solidified pads of 5% agarose laid on the slides. After adding a coverslip, worms were observed using a BX-51 microscope (Olympus).

### Sarcomere imaging

*Myo-3(st386); stEx30 [myo-3p::GFP::myo-3* + *rol-6(su1006)]*,which has GFP fusion MYO-3 (myosin) protein, was used for this study. For synchronized worms, eggs were collected by bleaching Tg animals and reared at 20 °C on OP50 normally-seeded NGM plates (Day 0). After 36 h, the worms were transferred to NGM plates with FBX (0, 5 or 10 µg/ml) and Km. UV-irradiated OP50 was used as food. After Day 4, we replated every day until the worm stopped laying eggs. On Days 12 after bleaching, worms were anesthetized by placing M9 buffer with a drop of 50 mM sodium azide on the solidified pads of 5% agarose laid on the slides. After adding a coverslip, body wall muscle cell VL quadrants 15, 17, VR quadrants 15 and 17 were observed using a BX-51 microscope (Olympus).

### Movement analyses

Eggs were collected by bleaching nematodes reared at 20 °C on OP50 normally seeded NGM plates (Day 0). After 36 h, bleached nematodes were transferred to NGM plates with FBX, uric acid, prednisone or solvent. OP50 was irradiated with UV and treated with Km. When the nematodes reached the young adult stage, FUdR (15 μM) was added to the NGM plate. Eleven days after bleaching, nematodes were placed on new NGM plates with one animal per plate. After 30 min, we photographed the traces of worm movement using a stereomicroscope (Olympus). The areas with worm movement were quantified using ImageJ (NIH, Bethesda, MD). At least 30 animals were observed per condition at a time. The experiments were repeated three times. Prednisone was dissolved and diluted in ß-cyclodextrin as previously described [16] and used at a final concentration of 1 mg/ml.

### Contraction assay

Each mutant animal was transferred to a fresh NGM plate (without bacterial seeding), and a movie was recorded for ten seconds. The movies were recorded at 0.07 s per frame. Movies in which the entire body of the nematode was captured continuously for more than three seconds were analyzed. The body size of the nematodes was measured for each frame of the movie using ImageJ (NIH, Bethesda, MD), and the average body size was calculated. The value of "body size (each frame)" minus "average body size" was calculated and plotted. The number of downward peaks was defined as the number of contractions. The rate of change in body area was calculated as the average of the subtracted values between the upper and lower peaks/number of contractions. Frequency of contraction/sec. was calculated as follows: number of contractions/number of analysis frames X 0.07 (Additional file 1: Fig. S3A).

### Mitochondrial and nuclear imaging.

*ccIs4251 (Pmyo-3::Ngfp-lacZ; Pmyo-3::Mtgfp)*, which has GFP fusion proteins localized to the body wall muscle mitochondria and nuclei, was used for this study. For synchronized worms, eggs were collected by bleaching *ccIs4251* and *dys-1(tm4402); ccIs4251* worms and reared at 20 °C on OP50 normally seeded NGM plates (Day 0). After 36 h, the worms were transferred to NGM plates with FBX (0, 5, or 10 µg/ml) and Km. UV-irradiated OP50 was used as food. When the nematodes reached the young adult stage, FUdR (15 μM) was added to the NGM plate.

On Day 12 after bleaching, worms were photographed using a szx12 microscope (Olympus) at 11.5 × magnification. The fluorescence intensities were quantified using ImageJ (NIH, Bethesda, MD).

### Statistical analysis

All data are presented as the mean ± SEM. For multiple comparisons, one-way ANOVA followed by Tukey’s test was used to compare groups. All tests were performed using GraphPad Prism version 6. All assays for drug effects were performed in double-blinded experiments. For all experiments, p values < 0.05 were considered to indicate significance.

## Results

### Administration of FBX suppresses C*. elegans* muscular dystrophy model phenotypes.

**Fig. 1:**
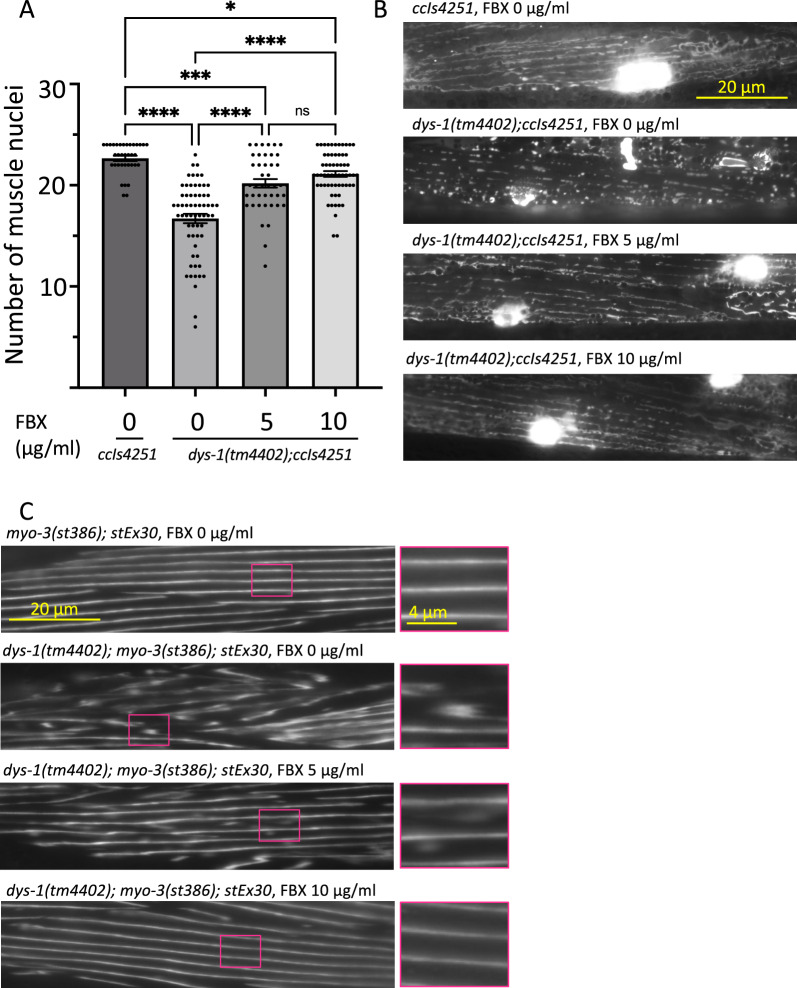
FBX has a protective effect on body wall muscle cells in *dys-1* mutant animals. **A** Wild-type and *dys-1* mutant animals were cultured on a medium containing FBX at the concentration indicated on abscissae, and the numbers of body wall muscle cell nuclei in one bundle per animal were counted on Days 12. *P < 0.05, ***P = 0.0006, ****P < 0.0001. **B** Representative images of wild-type and *dys-1* mutant animals’ mitochondria. Transgenic animals expressing mitoGFP in body wall muscle cells (*ccIs4251 [Pmyo-3::mitoGFP]*) were analyzed on Day 12 after bleach synchronization. **C** Representative images of wild-type and *dys-1* mutant animals’ myofilament. Transgenic animals expressing GFP fusion MYO-3 protein in body wall muscle cells were analyzed on Day 12 after bleach synchronization. Right column Fig. are 2.7-fold magnification of the boxed area

It has previously been shown that aging *dys-1(eg33)* mutant animals have greater mitochondrial fragmentation and myocyte death than wild-type animals [17, 18]. *dys-1* encodes a protein similar to the human dystrophin protein. In this study, we investigated a *dys-1* mutant allele, *dys-1(tm4402)*. The *tm4402* allele has a frameshift mutation near the 5' end in the *dys-1* gene (Additional file 1: Fig. S1A, B). We crossed *dys-1(tm4402)* animals with a transgenic strain, *ccIs4251 (Pmyo-3::Ngfp-lacZ; Pmyo-3::Mtgfp)*, which expresses GFP fusion proteins localized in the mitochondria and nuclei of body wall muscle cells. On Day 12 after bleaching, number of detectable GFP-labeled body wall muscle nuclei was counted. As described in a previous paper [19], *dys-1(tm4402)* similarly showed a reduction in the number of nuclei in the body wall muscle (Fig. 1A). The numbers of detectable GFP-labeled body wall muscle nuclei were increased in *dys-1(tm4402)* reared on a medium supplemented with FBX at 5 and 10 µg/ml than in those reared without FBX (Fig. 1A). The results indicated that FBX had a protective effect on the degeneration of *dys-1(tm4402)* body wall muscle cells.

We have previously reported that administration of FBX to wild-type *C. elegans* has a protective effect on myocytes and their mitochondria [10]. To test the effect of FBX on *dys-1(tm4402); ccIs4251*, this strain was grown on FBX (5 and 10 µg/ml)-supplemented medium, and mitochondrial fragmentation in the body wall muscle cells was observed on Day 12 after bleaching. In a previous paper, [18, 19], it was reported that *dys-1(cx18)* and *dys-1(eg33)* showed mitochondrial fragmentation. We also observed mitochondrial fragmentation in *dys-1(tm4402)* mutant animals. In contrast, FBX improved the mitochondrial fragmentation of *dys-1(tm4402)* mutant animals (Fig. 1B). The results indicated that FBX had a protective effect on the mitochondria of *dys-1(tm4402)* body wall muscle cells.

We measured fluorescence intensity in the whole body wall muscle cells. Wild-type animals showed strong GFP signals in the whole body wall muscle cells. In contrast, fluorescence in the muscle cells of the body wall was reduced in the *dys-1(tm4402)* mutant animals. FBX improved the fluorescence intensity in the whole body wall muscle cells of *dys-1(tm4402)* mutant animals.

However, the fluorescence intensity of FBX-treated *dys-1(tm4402)* mutant animals was weaker than that of wild-type animals (Additional file 1: Fig. S2A, B).

To investigate age-related sarcomere degeneration, we crossed *dys-1(tm4402)* animals with a transgenic strain, *myo-3(st386); stEx30 [myo-3p::GFP::myo-3* + *rol-6(su1006)]*, which expresses GFP fused MYO-3 (myosin heavy chain A) protein in body wall muscle cells. In wild-type animals on Day 12 after bleaching, 79% of animals (n = 71) were observed to have clear lines of sarcomere in all four cells (VL quadrants 15, 17, VR quadrants 15 and 17) (Fig. 1C). In *dys-1(tm4402)* mutant animals, sarcomere were misshaped, not properly aligned and lines of sarcomere were sometimes interrupted (Fig. 1C). In *dys-1(tm4402)* mutant animals, 0.4% of animals (n = 45) were observed to have clear lines of sarcomere in all four cells. FBX (10 µg/ml) improved the sarcomere degeneration of *dys-1(tm4402)* mutant animals (Fig. 1C). Sixty nine % of animals (n = 52) were observed to have clear lines of sarcomere in FBX (10 µg/ml) treated *dys-1(tm4402)* mutant animals. FBX (5 µg/ml) weakly improved the sarcomere degeneration of *dys-1(tm4402)* mutant animals (Fig. 1C). Fifty one % of animals (n = 56) were observed to have clear lines of sarcomere in FBX (5 µg/ml) treated *dys-1(tm4402)* mutant animals. The results indicated that FBX had a protective effect on the sarcomere degeneration of *dys-1(tm4402)* body wall muscle cells.

### *dys-1(tm4402); unc-22(e66)* is a model for age-related muscle weakness

As a progressive loss of locomotion was not clearly observed in *dys-1(tm4402)* mutant animals (data not shown), we investigated whether *dys-1(tm4402); unc-22(e66)* double mutant animals show a progressive loss of locomotion.

**Fig. 2:**
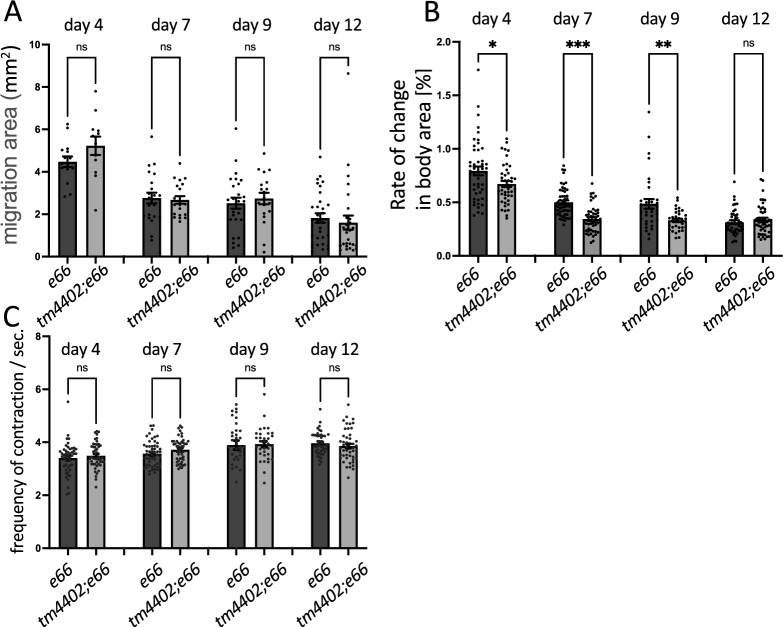
Weak contraction of the body wall muscles in *dys-1* mutant animals. *dys-1(tm4402); unc-22(e66)* and *unc-22(e66)* were grown synchronously at 20˚C and were observed at days 4, 7, 9, and 12. The distance the worms traveled in 30 min was quantified (**A**) Body wall muscle contractility was compared between *dys-1(tm4402); unc-22(e66)* and *unc-22(e66)*. Compared with *unc-22(e66)*, *dys-1(tm4402); unc-22(e66)* has weakly contracting body wall muscles. This effect of dystrophin depletion on muscle contractility was detected beginning on day 4 (at L4 stage) (**B**). Body wall muscle contraction rate was compared between *dys-1(tm4402); unc-22(e66)* and *unc-22(e66)* (**C**). *P = 0.231, **P = 0.009, ***P = 0.0002, *ns* not significant

UNC-22, also known as twitchin, is an invertebrate-specific protein, which may serve the function of the A-band portion of vertebrate titin, and/or vertebrate myosin binding protein C [20], a molecule involved in the excitation–contraction cascade of body wall muscle cells. Titin is known to be degraded when muscle is damaged. Titin degradation is remarkable in the skeletal muscles of muscular dystrophy patients. We used *unc-22(e66)* mutant animals to enhance the process by which formed body wall muscle cells are damaged by movement. We measured the area of movement of *dys-1(tm4402); unc-22(e66)* and *unc-22(e66)* mutant animals over 30 min at 4, 7, 9 and 12 days after bleaching. We found that the locomotory functions of both *dys-1(tm4402); unc-22(e66)* and *unc-22(e66)* mutant animals progressively declined with age. This decline did not differ between the two strains (Fig. 2A). We then measured body contraction in *dys-1(tm4402); unc-22(e66)* and *unc-22(e66)* mutant animals, as *unc-22(e66)* animals display the twitching phenotype (Waterston et al.). *dys-1(tm4402); unc-22(e66)* mutant animals showed weaker muscle contractility than *unc-22(e66)* mutant animals at 4, 7, and 9 days after bleaching (Fig. 2B, Additional file 1: Fig. S3, Additional file 1: Movies 1–8). These results are similar to those obtained in a previous study measuring muscle force in WT and *dys-1(eg33)* worms using NemaFlex [18]. Since the *unc-22(e66)* mutant is known to show a faster contraction–relaxation rate than the wild type [21], we compared the frequency of muscle contraction between *dys-1(tm4402); unc-22(e66)* and *unc-22(e66)* mutants. No difference in the frequency of muscle contraction was found between these two strains (Fig. 2C, Additional file 1: Fig. S3, Additional file 2: Movies S1-8). We have shown that alterations in muscle contractility in *dys-1* mutant animals can be detected by using an *unc-22(e66)* background.

### Coadministration of FBX and uric acid improves behavioral deficits in *dys-1(tm4402); unc-22(e66)* worms

**Fig. 3:**
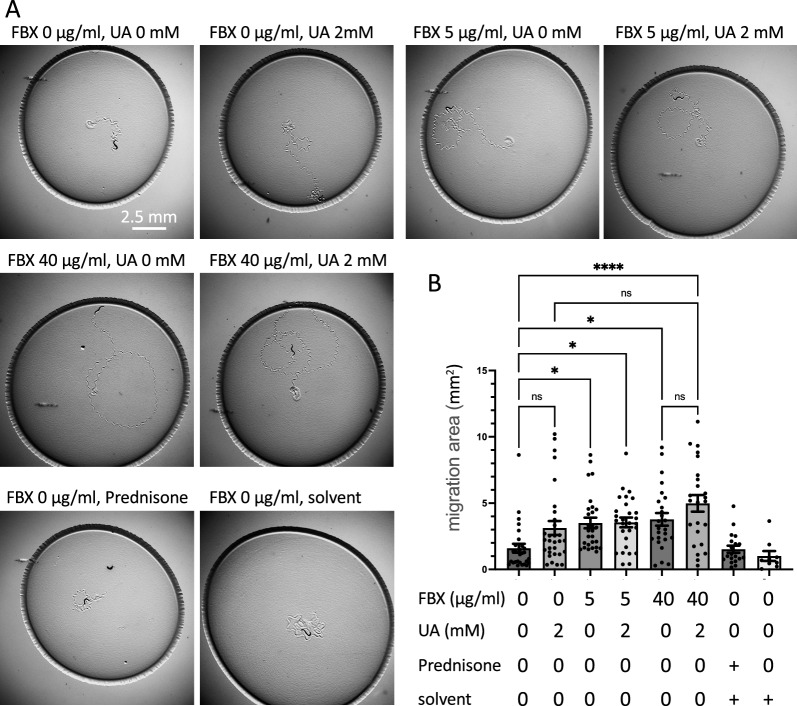
Mobility is maintained by co-administering FBX and uric acid in *dys-1(tm4402); unc-22(e66)* mutant animals. FBX (0, 5, 40 µg/ml), prednisone and uric acid (UA, 0, 2 mM) were added to *dys-1(tm4402); unc-22(e66)* mutant animals. twelve days after bleaching, nematode was placed on a new NGM plate with one animal each. After 30 min, the traces of worm movement were photographed. Representative images of worm tracking (**A**). The distance the worms traveled in 30 min was quantified (**B**). *P < 0.05, **** P < 0.0001. *ns* not significant

We showed that FBX suppresses mitochondrial damage in *dys-1* mutants (Fig. 1B). In addition, we have previously reported that coadministration of an antioxidant with FBX further enhances the effects of FBX [10]. Since weak muscle contractility was observed in *dys-1(tm4402); unc-22(e66)* worms (Fig. 2), FBX (0, 5, 40 µg/ml) and uric acid (0, 2 mM) were administered to *dys-1(tm4402); unc-22(e66)* animals, and the area of nematode movement over 30 min was examined at 12 days after bleaching. The migration distance of the nematodes was prolonged in the presence of FBX (5 and 40 µg/ml (Fig. 3A, B). In addition, coadministration of 40 µg/ml FBX and 2 mM uric acid further increased the migration distance of the animals (Fig. 3A, B). To determine whether FBX influences only *dys-1* mutant phenotype, we observed the effect of FBX on *unc-22(e66)* mutant animals (Additional file 1: Fig. S4). The area of nematode migration was measured and there was no significant difference between *dys-1(tm4402); unc-22(e66)* (0 µg/ml FBX) and *unc-22(e66)* FBX (0 µg/ml FBX) (Fig. 3B, Additional file 1: Fig. S4). Furthermore, the supplementation of FBX to *unc-22(e66)* had no apparent effect. This suggests that *unc-22* single mutation also causes a progressive decline in locomotion (Additional file 1: Fig. S4), although, FBX mainly works against the *dys-1* mutant phenotype.

Previous studies have examined the thrashing rate of dystrophin-deficient worms when placed in a liquid environment to determine their locomotor disorders. The thrashing rate of the *dys-1* mutant animals was lower than that of wild-type animals. This phenotype was suppressed by prednisone administration [18]. We administered prednisone to *dys-1(tm4402); unc-22(e66)* animals but did not observe any beneficial effect of prednisone in our behavior assay (Fig. 3A, B).

## Discussion

DMD is a severe progressive muscle disease caused by mutations in the gene that codes for dystrophin. In humans, *C. elegans* and mice, loss of dystrophin causes Ca^2+^ overload in muscle cells and mitochondria, mitochondrial damage, reduced ATP production and increased ROS release upon muscle contraction [8, 22, 23]. Finally, it causes inflammation and necrosis of skeletal muscle [24].

In the *dys-1(tm4402)* mutant animals, supplementation with FBX slightly improved the mitochondrial structure of body wall muscle cells (Fig. 1B), and coadministration of FBX and uric acid restored motility in *C. elegans* (Fig. 3).

These results suggest that FBX exerts a protective effect on mitochondria and body wall muscle cells in *dys-1* mutants by activating the salvage pathway of purine metabolism and supporting ATP production (Fig. 1), [10]. On the other hand, when FBX activates the salvage pathway of purine metabolism, the concentration of uric acid (antioxidants) decreases [10]. *dys-1* mutant animals have a higher basal oxygen consumption rate than the wild-type animals [18] and produce high levels of ROS. Therefore, coadministration of FBX and uric acid (antioxidant) was more effective than administration of FBX alone (Fig. 3). Prednisone administration, NaGYY administration and mcu-1 inhibition in *dys-1* mutant animals are known to improve fragmented mitochondrial networks and restore motility [16, 25, 26], but these points of action differ from those of FBX.

Muscles show an increase in cytosolic Ca^2+^ concentration with contraction. *unc-22* mutant animals are known to exhibit a phenotype of increased frequency of contraction and relaxation [21]. Therefore, the *dys-1; unc-22* double mutant animals may have had an enhanced increase in cytosolic Ca^2+^ concentration during muscle contraction, a common early pathology of DMD, making the *dys-1* mutant phenotype easier to observe (Figs. 2, 3, Additional file 1: Fig. S3, Additional file 2: Movies S1-8). There was no difference in contraction frequency between *dys-1; unc-22* double mutant animals and *unc-22* mutant animals, but contraction strength was reduced in the presence of *dys-1* mutation. Progressive muscle weakness is known to be present in human DMD beginning in childhood [27], and a similar pathology was observed in *dys-1; unc-22* double mutant animals.

In addition to skeletal muscle symptoms, other cranial neurological symptoms, such as cognitive impairment and learning disability, are also observed in DMD [28]. The incidence of autism in patients with DMD is known to be higher than that in controls [2, 28]. Dystrophin is expressed in the central nervous system as well as in muscles [29]; therefore, administration of FBX may also increase ATP levels in the central nervous system and alleviate cranial nerve symptoms. FBX inhibits XOR (an enzyme that converts hypoxanthine to uric acid) and increases plasma hypoxanthine levels [30]. Hypoxanthine passes the blood‒brain barrier [31], and hypoxanthine is converted to ATP by the purine salvage pathway [32].

At present, there is no radical therapy for DMD. The main approved treatment is the administration of prednisone [5]. Exon-skipping therapies are in development but are indicated for a limited number of patients [9]. Therefore, there is a need to develop drugs that target the common initial pathology of DMD. Treatment for muscle symptoms of DMD needs to prevent or delay muscle necrosis until dystrophin replacement therapy is available, and FBX is a candidate drug for this purpose. Future studies are needed to determine whether FBX is effective as a treatment for the central nervous system symptoms of DMD.

### Supplementary Information

**Additional file 1: Fig. S1.*** C. elegans* mutant of *dys-1*. A, schematic diagram of the *dys-1* gene and their mutants. B, The structures of human dystrophin and *C. elegans* DYS-1. The key motifs of human dystrophin and *C. elegans* DYS-1 are almost equivalent. **Fig. S2.** FBX slightly suppresses the decrease in overall muscle fluorescence in* dys-1* mutant animals. A, Representative images of fluorescence intensity. wild-type (top) and *dys-1* mutant animals (Bottom three pictures) expressing GFP in the muscle nuclei and mitochondria (*ccIs4251[Pmyo-3::Ngfp-lacZ; Pmyo-3::Mtgfp*]) were grown synchronously at 20 °C and were observed at day 12. *dys-1* mutant animals were cultured on a medium containing FBX at the concentration indicated on abscissae. B. Fluorescence intensity quantified using ImageJ. At least 12 nematodes were observed in each condition. *P =0.03, ns, not significant. **Fig. S3.** Quantitative analysis of the body wall muscle contraction and relaxation assay. A, Schematic diagram for the contraction assay. Graphs showing the body wall muscle contraction and relaxation processes of (B) *unc-22(e66)* and (C) *dys-1(tm4402); unc-22(e66)* mutant animals at days 4, 7, 9, and 12. **Fig. S4.** Mobility is maintained by co-administering FBX and uric acid in *unc-22(e66)* mutant animals. FBX (0, 5, 40 µg/ml), prednisone and uric acid (UA, 0, 2 mM) were added to *unc-22(e66)* mutant animals. twelve days after bleaching, nematode was placed on a new NGM plate with one animal each. After 30 min, the traces of worm movement were photographed. The distance the worms traveled in 30 minutes was quantified. *P= 0.0451.

**Additional file 2: Movie S1.** Time lapse video of *unc-22(e66)* mutant animal at day 4 after bleaching. The movie corresponds to Additional file 1: Fig. S3B. **Movie S2.** Time lapse video of *unc-22(e66)* mutant animal at day 7 after bleaching. The movie corresponds to Additional file 1: Fig. S3B. **Movie S3.** Time lapse video of *unc-22(e66)* mutant animal at day 9 after bleaching. The movie corresponds to Additional file 1: Fig. 3B. **Movie S4.** Time lapse video of *unc-22(e66)* mutant animal at day 12 after bleaching. The movie corresponds to Additional file 1: Fig. S3B. **Movie S5.** Time lapse video of *dys-1(tm4402); unc-22(e66)* mutant animal at day 4 after bleaching. The movie corresponds to Additional file 1: Fig. S3C. **Movie S6.** Time lapse video of *dys-1(tm4402); unc-22(e66)* mutant animal at day 7 after bleaching. The movie corresponds to to Additional file 1: Fig. S3C. **Movie S7.** Time lapse video of *dys-1(tm4402); unc-22(e66)* mutant animal at day 9 after bleaching. The movie corresponds to to Additional file 1: Fig. S3C. **Movie S8.** Time lapse video of *dys-1(tm4402); unc-22(e66)* mutant animal at day 12 after bleaching. The movie corresponds to to Additional file 1: Fig. S3C.

## Data Availability

The datasets used and analyzed during the current study are available from the corresponding author on reasonable request.
