# Supplementary material for: Febuxostat ameliorates muscle degeneration and movement disorder of the dystrophin mutant model in Caenorhabditis elegans
Source: J Physiol Sci. 2023 Nov 10;73:28. doi: 10.1186/s12576-023-00888-y (PMC10717159; doi:10.1186/s12576-023-00888-y)
Supplement: Supplementary file 1 — Additional file 1: Fig. S1. C. elegans mutant of dys-1. A, schematic diagram of the dys-1 gene and their mutants. B, The structures of human dystrophin and C. elegans DYS-1. The key motifs of human dystrophin and C. elegans DYS-1 are almost equivalent. Fig. S2. FBX slightly suppresses the decrease in overall muscle fluorescence in dys-1 mutant animals. A, Representative images of fluorescence intensity. wild-type (top) and dys-1 mutant animals (Bottom three pictures) expressing GFP in the muscle nuclei and mitochondria (ccIs4251[Pmyo-3::Ngfp-lacZ; Pmyo-3::Mtgfp]) were grown synchronously at 20 °C and were observed at day 12. dys-1 mutant animals were cultured on a medium containing FBX at the concentration indicated on abscissae. B. Fluorescence intensity quantified using ImageJ. At least 12 nematodes were observed in each condition. *P =0.03, ns, not significant. Fig. S3. Quantitative analysis of the body wall muscle contraction and relaxation assay. A, Schematic diagram for the contraction assay. Graphs showing the body wall muscle contraction and relaxation processes of (B) unc-22(e66) and (C) dys-1(tm4402); unc-22(e66) mutant animals at days 4, 7, 9, and 12. Fig. S4. Mobility is maintained by co-administering FBX and uric acid in unc-22(e66) mutant animals. FBX (0, 5, 40 µg/ml), prednisone and uric acid (UA, 0, 2 mM) were added to unc-22(e66) mutant animals. twelve days after bleaching, nematode was placed on a new NGM plate with one animal each. After 30 min, the traces of worm movement were photographed. The distance the worms traveled in 30 minutes was quantified. *P= 0.0451. [file 12576_2023_888_MOESM1_ESM.pdf]

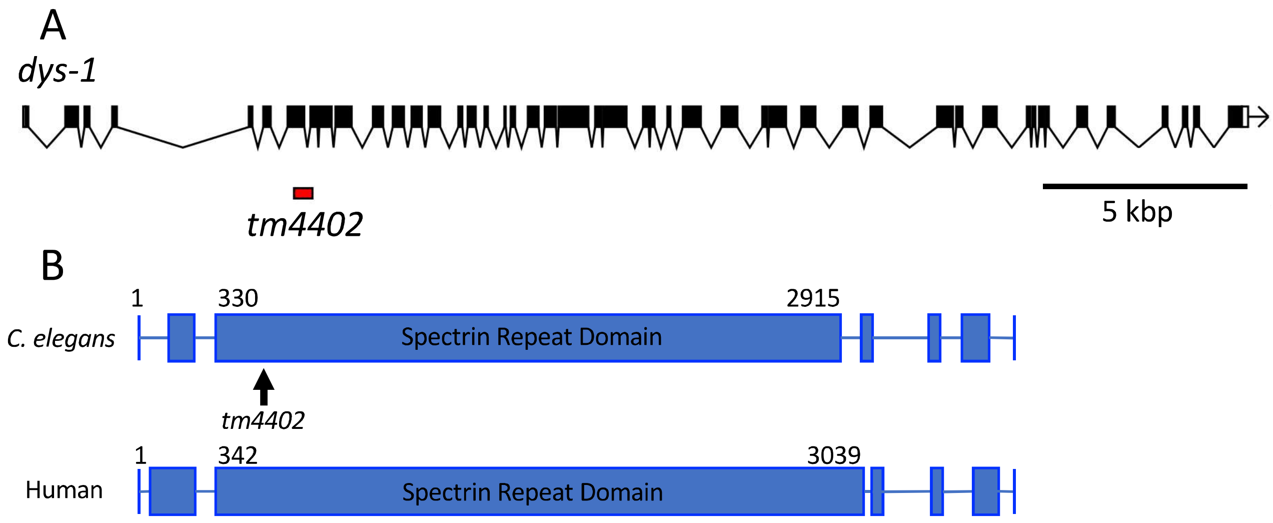

supplementary Fig. 1

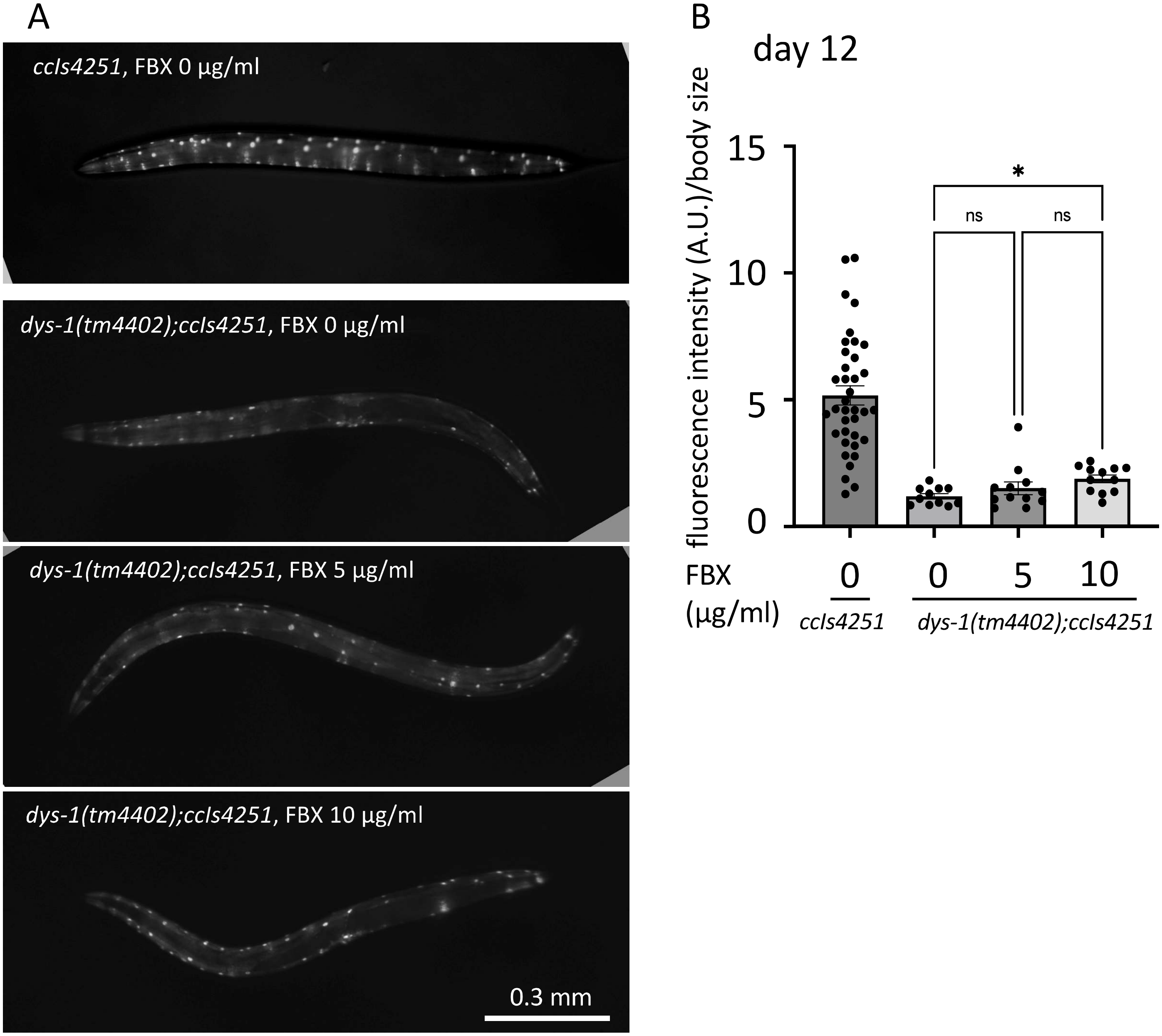

supplementary Fig. 2

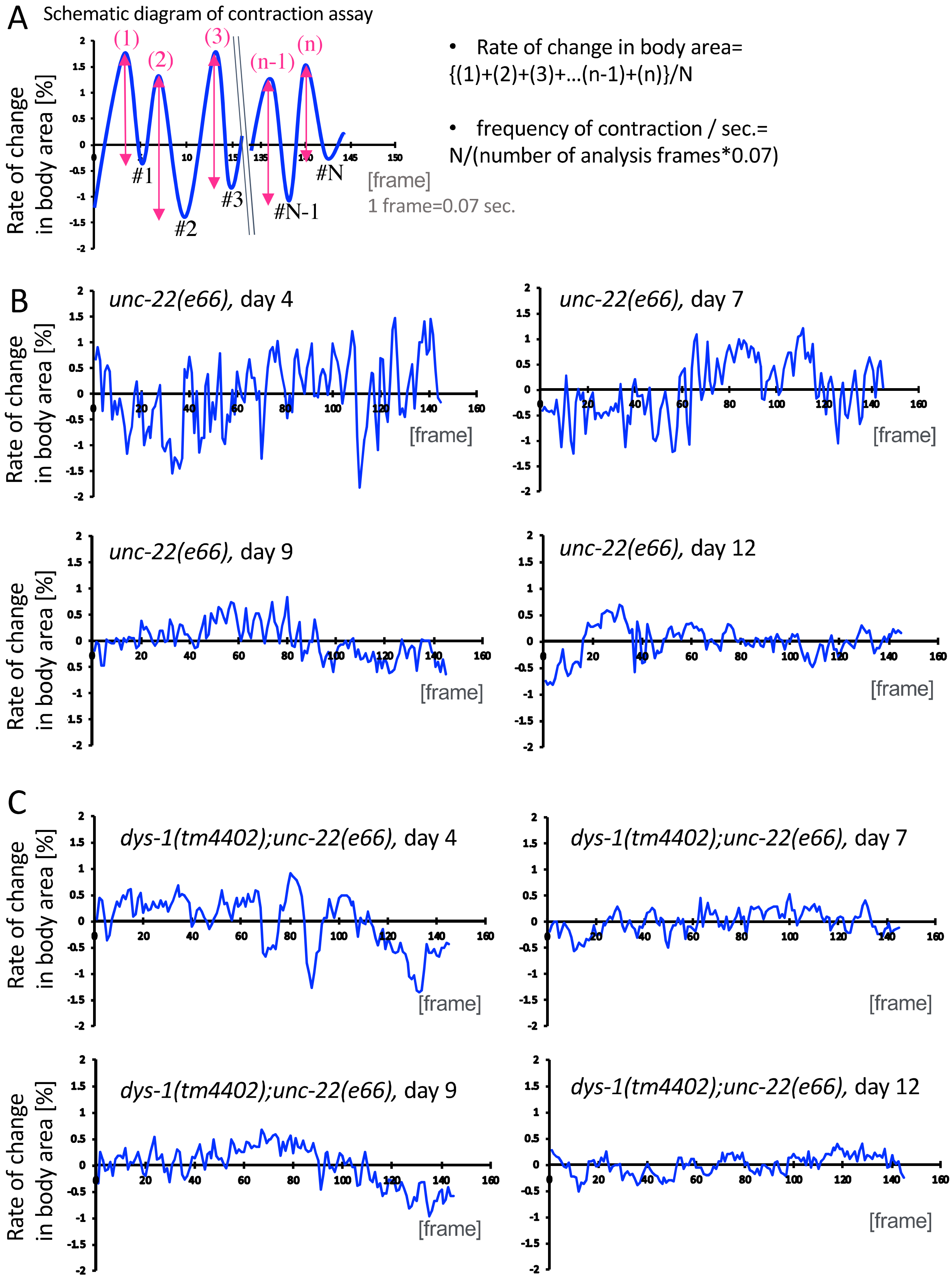

supplementary Fig. 3

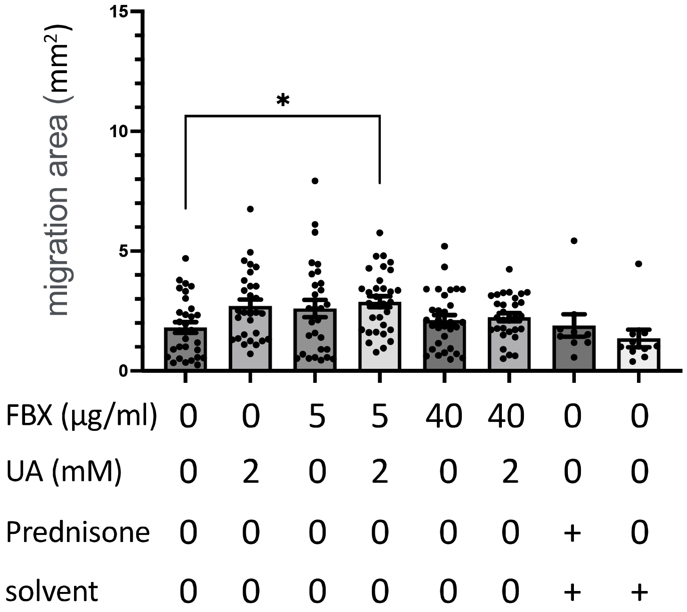

supplementary Fig. 4
